# Preferences, Needs, and Values of Patients With Chronic Obstructive Pulmonary Disease Attending a Telehealth Service: Qualitative Interview Study

**DOI:** 10.2196/53131

**Published:** 2024-06-21

**Authors:** Camilla Wong Schmidt, Karen Borgnakke, Anne Frølich, Lars Kayser

**Affiliations:** 1 Medical Department Holbæk Sygehus Region Zealand Holbæk Denmark; 2 Section of Health Services Research University of Copenhagen Copenhagen Denmark; 3 Innovation and Research Centre for Multimorbidity Slagelse Hospital Region Zealand Slagelse Denmark; 4 Department of Public Health University of Copenhagen Copenhagen Denmark

**Keywords:** people with long-term health condition, patient education, COPD, digital health, ethnography, inductive, ethnographic, chronic, lung, lungs, pulmonary, respiratory, self-management, interview, interviews, qualitative, experience, experiences, attitude, attitudes, opinion, perception, perceptions, perspective, perspectives, acceptance

## Abstract

**Background:**

Digitally assisted health care services and technologies are gaining popularity. They assist patients in managing their conditions, thereby reducing the burden on health care staff. Digital health care enables individuals to receive care that is more tailored to their needs and preferences. When implemented properly, it can promote equity by considering each person’s opportunities and limitations in the context of health care needs, preferences, values, and capabilities.

**Objective:**

This study aims to understand the needs, values, and preferences of individuals with chronic obstructive pulmonary disease (COPD) who are provided with a 24/7 digital health care service. Furthermore, we aim to understand the dynamics of the communities to which they belong and how these communities intersect. This will provide us with the essential knowledge to establish new methods of providing education, including the development of educational activities for health professionals to engage, train, and empower people living with COPD.

**Methods:**

The study included 7 informants diagnosed with COPD who received 24/7 digital health care service support from a regional project in Region Zealand, Denmark. The informants were visited 4 times during 2 months, including a “Hello” visit, a day with a semistructured interview, and 2 days with field observations. The informants participated in a semistructured interview, following participant observation and an ethnographic approach. The interview content was analyzed using an inductive methodology to categorize the empirical data.

**Results:**

Using the inductive approach, we identified 3 main categories related to the informants’ needs, values, and preferences: (1) Health, (2) Value Creation, and (3) Resources. These 3 main categories were based on 9 subcategories: (1) health and barriers, (2) self-monitoring, (3) medication, (4) behavior, (5) motivation, (6) hobbies, (7) social networks, (8) health professionals, and (9) technology. These findings revealed that the informants placed value on maintaining their daily activities and preserving their sense of identity before the onset of COPD. Furthermore, they expressed a desire not to be defined by their COPD, as conversations about COPD often shifted away from the topic.

**Conclusions:**

Digital health solutions and the health care professionals who offer them should prioritize the individuals they serve, considering their needs, values, and preferences rather than solely focusing on the medical condition. This approach ensures the highest level of daily living and empowerment for those living with long-term health conditions. The communities surrounding individuals must engage in constant interaction and collaboration. They should work together to incorporate people’s needs, values, and preferences into future digital health services, thereby promoting empowerment and self-management. New educational programs aimed at developing the digital health service competencies of registered nurses should facilitate collaboration between the 2 communities. This collaboration is essential for supporting patients with long-term health conditions in their daily activities.

## Introduction

### Background

In recent years, a transformation has been occurring with the increased use of digitally assisted health care services and technologies. These advancements aim to reduce the burden on the health care work force by enabling patients to better manage their own conditions [1]. Digitally based health care offers an opportunity for personalized and tailored health care services that better meet the needs of individual patients. Digitalization can also help reduce inequities if introduced thoughtfully, with an awareness of both the opportunities and barriers for individuals, considering their health care needs, preferences, values, and capabilities [1].

When health care professionals have the appropriate knowledge about these factors and are trained to address them, it can facilitate meaningful conversations and better connections with those they serve. This, in turn, can increase patient motivation and ease their access to digital services [2,3]. This necessitates educational programs for health professionals that focus on understanding their patient’s needs and values, capability, person-centered services, self-management, and communities surrounding the patient. These programs should be based on evidence from empirical data obtained through interviews and observations of individuals with firsthand experience using digitally enabled health care services [4].

### Digital Health Care and Chronic Obstructive Pulmonary Disease

Chronic obstructive pulmonary disease (COPD) is a leading cause of death, responsible for over 3 million deaths worldwide in 2019 [5]. In Denmark, 3355 individuals died from COPD in 2020, making it the second most common cause of death in the nation [6]. As a result of the progressive loss of pulmonary function, people with COPD experience impairments in their daily activities. These impairments can inhibit mobility, leading to a sedentary lifestyle [7]. The Global Initiative for Chronic Obstructive Lung Disease (GOLD), established in 1998, has developed a set of recommendations for managing COPD. Evidence shows that self-management improves outcomes for patients with COPD and reduces the likelihood of hospitalization [8,9]. The recommendations by GOLD also address how increased self-management can help motivate and engage people, leading to positive adaptations in their health behaviors.

Digital health monitoring is one of the most widely used tools for self-management of COPD and other long-term health conditions (LTHCs) and appears to reduce the risk of both hospitalization and acute visits [10]. In 2015, a randomized controlled study was conducted with 100 people with COPD, with 48 randomly selected for home monitoring and 52 for usual care. The study found that people with COPD who used a home monitoring kit for 6 months had improved health-related quality of life and reduced anxiety scores compared with people who received usual care. The study also showed that people with home monitoring kits had fewer and shorter hospitalizations than those receiving usual care [11]. Furthermore, people with COPD who participate in telemedicine-based interventions feel safer, more empowered, and more in control of their own disease [11].

People with LTHCs who possess an enhanced ability to manage their condition themselves and experience a higher level of empowerment tend to have a reduced risk for hospitalization [12]. A likely explanation for this can be found in a qualitative study from 2017, which identified 3 main themes for individuals participating in a telemedicine intervention: (1) a sense of improved security and control; (2) a better understanding of their disease; and (3) the benefits of virtual conversations [13]. However, these studies do not take place within person-centered digital health communities and do not address the significance of the participants’ sociodemographic characteristics. Evidence of sociodemographic characteristics is important, as research indicates that resourceful individuals benefit more from available health care, and those with an interest in technology may derive greater benefits from digital health solutions [14]. In addition, the prevalence of COPD and other chronic conditions, such as ischemic heart disease and type 2 diabetes mellitus, is higher in areas with populations characterized by lower sociodemographic status than in the average population [15,16]. This may contribute to the risk of inequity, as lower sociodemographic status often correlates with both lower levels of education and lower digital health literacy [15,17].

### Educational Programs for the Digital Transformation

Another problem often overlooked concerning disadvantaged individuals living with 1 or more LTHCs is their reluctance to attend traditional educational services structured around scholastic planning. To reduce inequity, there is a need to develop new approaches to include this segment of the population, utilizing educational methods that are not scholastic or built on classical teaching methods such as classroom-based education. In response to this need, we have initiated a project where, based on an ethnographic approach involving interviews, observations, and the cocreation of educational materials, we will develop a new methodology inspired by social learning theory [18,19]. To provide guidance for the development of new educational programs and curricula tailored for digital transformation, we have examined, on an individual level, the preferences, values, and needs of people with COPD in the context of unrestricted access to a person-centered, digitally enabled health service available 24/7. Informed by social learning theories, we have also explored the existence of communities these individuals are part of in relation to their everyday lives and their ability to identify potential sources of support.

The purpose of this study is to gather essential information about individuals living with COPD within the context of accessing support from a regular 24/7 digital health care service and the dynamics of the communities to which they belong. This information will empower us to design new methods for providing education, including the development of educational activities for health professionals. These activities will enable professionals to effectively engage, train, and empower people with COPD.

This has led us to the following research questions:

- Research question 1: What matters for people with COPD with respect to their needs, values, and preferences in the context of using a 24/7 digital health service?
- Research question 2: What is the role of the health care and social networks, respectively, and how are these a potential part of communities formed around the patient’s health condition?

## Methods

### Design

This report is a part of a larger PhD project and constitutes the first of 4 articles. The overarching aim of this work is to obtain insights into the lives of people with COPD, supported by a 24/7 digital health service, and to use this information to develop a patient-case–based curriculum to educate health professionals on effectively engaging, enabling, and empowering individuals living with COPD.

The first study reported here was conducted from August 1, 2020, to January 31, 2021. This period coincided with the COVID-19 pandemic in Denmark before vaccinations were introduced to protect against severe cases. The study is qualitative in nature, inspired by ethnographic research methods, and includes semistructured interviews and field observations [20]. The field study involved visiting the homes of people with COPD 4 times over 2 months. The visits included a preliminary “Hello” visit, a day dedicated to a semistructured interview, and 2 days focused on field observations. This article focuses on the data and results gathered during the second visit, which involved conducting the semistructured interview.

### Context

The digital health service utilized in this study is provided by an innovation project in Region Zealand, Denmark, called PreCare. This project is built on the Epital Care Model (ECM) [21,22]. The ECM, developed in 2016, offers a 24/7 digital health care service where individuals with LTHCs monitor their own health with the assistance of nurses from a response and coordination center (RCC). The ECM consists of 6 stages: citizens with unknown LTHCs, active and independent living, virtual assisted living, virtual assisted living with support from health care professionals, outpatient care at home, and admission to a local health clinic or hospital. It serves as a template for digital health services based on patients’ medical needs [22].

In total, approximately 400 participants with COPD or ischemic heart disease were enrolled in the PreCare project over a 4-year period. At any given time, there are approximately 150 participants. Each participant was provided with a tele-home monitoring kit, which included a tablet. Additionally, for participants with COPD, the kit included a spirometer, a thermometer, a pulse oximeter, and a box containing acute medicine. The participants were supported by an RCC, which was staffed with registered nurses (RNs) and an eDoctor. According to the project protocol, participants monitored their condition daily. They could always call the RCC to discuss their condition, and the RCC regularly initiated contact to ensure participants felt safe and confident. During these conversations, self-management was also supported (C Schmidt, MSc, personal communication, 2020). In the event of deterioration, the tablet would indicate a yellow or red code and send a message to the RCC. The nurses would then respond to the code and call the participant to follow-up on the reported condition. If needed, participants can take medicine from the box to treat exacerbations. In cases of further need, the nurses would contact the eDoctor [23].

### Informants

The PreCare project initially provided a list of 15 participants diagnosed with COPD, each with varying degrees of severity, all of whom expressed interest in participating in the research related to the PreCare project. Subsequently, over a 4-month period, 10 of these participants were contacted by phone, selected from the top of the list. After receiving oral information about the project, 8 of these agreed to participate. However, 2 participants were not interested and the remaining 5 were not contacted as the recruitment period had exceeded. Furthermore, 1 potential informant expressed disinterest in participating after the initial meeting. The selected number of informants was determined by the limitations of the study design. The informants were invited in 3 separate periods: 3 informants were invited from August to September for the first period, 2 informants were invited from October to November for the second period, and 2 more were invited from December to January for the third period. In total, 7 informants were recruited. After obtaining oral consent via phone, further information was sent by email to 3 informants, while the other 4 did not require this. After 1 week, all 7 informants were contacted again by phone to schedule the first in-person meeting, which took place within 1-2 weeks. This study’s inclusion and exclusion criteria followed the PreCare protocol [23].

### Data Collection

To establish an emotional and trustworthy relationship with the informants, we scheduled a visit to their homes (the initial visit). This approach aimed to strengthen the connection between the researcher and the informants, fostering an informal and friendly atmosphere during the interview. To conduct the semistructured interview, we used an interview guide inspired by Spradley’s [20] ethnographic interview techniques. The interview was conducted in a friendly and casual approach, allowing the informants to share their experiences and discuss their everyday lives with a chronic condition as they deemed appropriate [20,24]. All interviews were conducted in the informants’ homes and were audio-recorded with their consent. As a result of the informants’ background, the interviews were conducted in Danish, and only quotes were later translated into English by the first author (CWS). All informants participated in the interviews; 1 participant had his spouse present during the interview.

### Interview Guide

The interview guide was developed based on sociotechnical ecosystem thinking, our concept of technology readiness, and an attempt to identify how individuals belong to 1 or more communities, inspired by social learning theory (Multimedia Appendix 1) [25-28]. We conducted the interview with an open-minded approach, including “how” questions, to enable the informants to respond as they found suitable. The interview guide was structured around 6 thematic areas: daily activities, health, measurements, communities, RCC and PreCare, and literacies and digital literacies. For each of the thematic areas, we included 1 main question and underlying questions to sustain the conversation throughout the interview. For example, the theme “daily activities” included the main question: “Can you tell me how a typical day is for you?” In the theme “health,” the main question was “Can you tell me how COPD has affected your life?.” The 6 themes were defined by the authors and were written in Danish.

### Data Analysis

The interviews were conducted, transcribed, and analyzed by the author CWS. The transcripts were analyzed using content analysis, a method for systematically and objectively describing and quantifying phenomena. An inductive approach was used, beginning with open coding to create categories, followed by abstraction to generate main categories [29]. A 3-step content analysis was used to identify the main categories.

### Analysis of Interviews

Each interview was transcribed and carefully reviewed to understand the context of the data. Subsequently, the transcripts were uploaded and coded using NVivo 12 (QSR International) [30] by CWS. Over 700 codes were identified and categorized into 66 subcategories. These subcategories were then merged to create an affinity diagram initially using paper and later repeated using NVivo. This process resulted in 9 categories, each containing 4-12 subcategories, respectively. The category “Self-Monitoring” had the fewest subcategories, while “Health Professionals and Social Network” had the most subcategories. The 9 categories were analyzed by CWS and the last author (LK) to synthesize the data into 3 main categories. CWS, who holds an MSc degree in health informatics and has been educated in qualitative methods, collaborated with LK, a professor in health service research with experience in both qualitative and quantitative analyses, for this process.

The 3 main categories identified were health, value creation, and resources (Textbox 1 and Multimedia Appendix 2). The category of health consisted of 3 subcategories: health and barriers, medication, and measurements. The category of value creation was formed from hobbies, behavior, and motivation. The category of resources was merged from 3 subcategories: social networks, health professionals, and technology. In our analysis, we paid particular attention to what matters to people with COPD, supported by the theories upon which the interview guide was built.

**Overview of the 3 main categories and subcategories.**

**1. Health**

- Health and barriers
- Self-monitoring
- Medication

**2. Value Creation**

- Behavior
- Motivations
- Hobbies

**3. Resources**

- Social network
- Health professionals
- Technology

### Ethical Consideration

Information regarding the study, partnerships, and data handling complies with the Helsinki Declaration and was communicated to the informants in both written and oral forms. They were informed that their participation was voluntary and anonymous and that they could revoke their consent at any time. Furthermore, they were assured that their involvement would not prevent them from participating in the PreCare project. All consent was obtained before the interview, through the signing of a consent form. The Danish National Center for Ethics was not required to approve the study as no biological material was used. Any data obtained from the informants were treated as personal health information and handled in accordance with Danish legislation (General Data Protection Regulation [GDPR]) and securely stored on drives. Health science questionnaire surveys and interview studies that do not involve human biological material [section 14(2) of the Danish Act on Committees] do not require reporting or approval from the Danish National Centre for Ethics [31].

## Results

### Characteristics of the Informants

A total of 4 men and 3 women participated in the interviews (age range 52-81 years). Two informants lived with their spouses. Despite having had COPD for an average of more than 2 years, the severity of each participant’s COPD varied. Some participants continued to smoke daily despite being aware of the health risks. One male participant was unable to provide information to categorize his level of education, 2 had only completed elementary school, while 4 had completed higher education. There was no evidence of their usage of technology, such as websites and participation in online communities, in relation to their medical concerns. All informants had been included in the PreCare Project for more than 6 months.

### The Three Main Categories

The main categories and subcategories identified in the content analysis provide insight into and offer a comprehensive understanding of the daily life situations and experiences of the informants living with COPD. Upon reviewing these categories, attention is drawn to both the specific consequences of a COPD diagnosis and how practical hurdles and activity levels are affected in the daily lives of the informants. These impacts are described in the interviews as limitations on activities the informants were accustomed to participating in, as well as a determination to carry out specific household duties despite a decreased energy level. The duality between “restrictions” and “experimental salvage” is evident in the category of activity but is also observed when interviews approach questions such as self-monitoring. Here, they take on different meanings, tasked with reclaiming self-discipline and control on one hand, while also being concerned that daily measurements can serve as a reminder of one’s limitations, akin to “being reminded of having a chronic disease.” Thus, through the interviews, it becomes apparent how the informants encounter difficulties and impediments in carrying out daily tasks due to their condition. In everyday life, this translates to tasks that were once feasible but now being difficult or impossible to complete. The distinction between “then” and “now” is frequently referenced, highlighting the contrast between the condition “before I got COPD” and “the situation as a chronic.”

### Health

#### Health and Barriers

This category describes the experience of living with COPD, detailing how it has impacted daily life and outlining the physical and mental barriers experienced throughout the day or in general.

The informants did not express interest in delving deeper into their everyday lives with COPD. Instead, they prioritized discussing other aspects of their daily life or past experiences. They responded quickly to questions about COPD and then redirected the conversation toward other topics. This deliberate redirection indicated their reluctance to discuss their chronic illness.

Interviewer: ...Can you tell us how your diagnosis has affected your life?

M3: So, I’m crushed. One positive thing is that I had to sell my motorcycle and all my stuffs, I used to gather a lot. We had a 400 kvm house with basement and ceiling, which was filled with enamel sign, books, magazines, tech cars and bicycles...

The informant swiftly and effectively shifts his focus away from negative thoughts about COPD’s interference and begins discussing his previous interest in used objects. He demonstrates a clear desire not to dwell on the negative aspects of his existence, opting instead to redirect the conversation toward something positive and reminiscent of happier times.

The limitations imposed by COPD forced the informants to forego certain daily activities, some of which could have contributed to an improved quality of life. The frequent shortness of breath and coughing prevented them from engaging in activities such as walking outside, performing household duties, or general personal care needs.

Interviewer: ...but is there other things COPD had done, that you cannot do anymore, completely?

M1: Well, I cannot go to the city and get me a cup of coffee at the street restaurant.

Interviewer: No, that’s true...

M1: I’m not even sure I’d be able to go to the garbage cans anymore (coughing), but when my friend comes and the weather is good (...) he drives me in that wheelchair over there, and then we sit together and drink a cup of coffee and talk...

The informant’s worry about his capacity to take out the trash underscores the profound impact that COPD has on his life, to the extent that he feels unable to leave the house without assistance. Conversations with the informant were replete with stories where social interactions played a significant role.

For some individuals, participating in a community became challenging due to shortness of breath caused by COPD. Additionally, for others, COPD had led to the complete exclusion of previous acquaintances.

M3 wife: In return, you have thought about how many of them you have helped (...), you don’t really hear from them anymore, because now you can’t help them anymore.

M3: Yes, there are many of those whom I have been calling, “Great that we are talking to you, we were just thinking of you, by the way we have a locker that doesn’t work”. You never heard from these people again, and I have been discussing this with others, and it is true...

The informant noted that his inability to visit friends anymore, coupled with their failure to reciprocate, has made it increasingly difficult for him to maintain relationships with them. This situation has surprised him, particularly because he is no longer able to provide assistance, as reported by his wife.

#### Self-Monitoring

This category highlights how the informants manage and self-monitor their condition. Furthermore, it explores how the outcomes of their monitoring efforts may impact their day and their motivation to engage in activities.

In the informants’ descriptions of their daily lives, the topic of self-monitoring for the PreCare project was not initially mentioned. It was only during the conversation around this subject that the activity itself was explained and, in some cases, mentioned.

M1: Yes but, it’s not interesting, no(...) and then I hope in the end it can help other people too. So, I take it with pleasure, but I could still think of something more exciting things to do...

W1: Yes, but they have changes it (pause), I’m just going to write something today, I’m not in for it. It’s not correct anyway (temperature)

The self-monitoring is described here as uninteresting, with 1 informant considering it a waste of time because he could find more engaging activities to do during his challenging day. The second informant emphasizes the importance of accurate measurements for individuals to actively participate in self-monitoring.

The outcomes of self-monitoring had a significant impact on the informants. The results were displayed as 1 of 3 colors—red, yellow, or green—on the tablet’s display. The meaning of the color had a tangible effect on the informant’s day.

Interviewer: Yes, exactly when, but then how? Because now you said that you had a red measurement yesterday was it then a difficult day when you have a red measurement?

W2: Yes, that is a stupid day, at first the mood is going down, and I am going, well yes I usually get restless, because I can’t, because a day like that, I am thinking about...is it now it’s going in the wrong direction...

The informant faces difficulties getting through the day when the red color appears, disrupting their daily routine. Additionally, the informant begins to question whether their COPD-related health is deteriorating or if the red color indicates a negative trend.

Conversely, the green color holds significance for the informant, particularly in contrast to the red color. Seeing a green measurement might enhance the informant’s enjoyment of daily life, especially if it has been a while since they last saw a green result.

W1: It’s green! (happy/excited)

W1: It haven been that for a long time....

Although there was considerable excitement surrounding the green measurement, its implications for the maintenance of the day remained unclear. However, the informant did clarify early in the conversation that she felt more motivated to venture outside into the garden on good days.

#### Medication

Being chronically ill entails the necessity of medication and its management which, for most of the informants, has become integrated into daily life. This category elucidates how the informants handle their medication and who supports them in managing it. The informants varied in their approaches to and understanding of medication, and the availability of help and support was crucial.

W2: Thus, those prescriptions, I also have one lying here, and this is the new medicine I got, and I don’t understand it, because I should have asked about it.

W3: Yes, I have these blue folders, you probably don’t know them, but those blue boxes for morning, midday, evening, and there is for (cough)...think there is for eight days, probably, that can be right? Eight days, I believe that, and I sort them every second week. I sit by the dining table, and line the whole thing up, and then I sort them.

The aforementioned examples depict 2 different scenarios of handling and understanding medicine. In the first scenario, one of the informants blames herself for not seeking information about the new medication when she first started taking it because she is unfamiliar with it. While she accepts responsibility for her medication, she still requires assistance in understanding it. By contrast, the second informant has established routines for managing her medication, and therefore, understands what she deposits into the pillbox.

As a result of errors and inconsistent care from municipal employees, the informants began to question their trust in the municipality’s care team. Although the informants could receive assistance from the municipality with their prescriptions and medication management, they found that the assistance and knowledge provided by municipal employees lacked the necessary qualifications.

W2: ...but she wasn’t, and then she made the mistake of repeating after the other, and I quickly notice it, and it’s not, it is not calcium tablets we sit and play with.

This is a serious concern, as indicated by the informant’s statement that the pills are not calcium supplements, and taking medication in incorrect amounts could have negative effects on her health. Therefore, it is vital that her medication is prescribed correctly for her condition.

When it comes to medication, there was a strong tendency among the informants to rely on the RCC nurses, especially during exacerbations. The RCC nurses use telephone communication to reach out to the informants and inquire about their health. If necessary, the nurses may advise the informants to take additional medication. The informants comply with the nursing advice and adjust their medication accordingly because they have a high level of trust in the digital nurses.

M2: Yes, “Nærklinikken” is the ones who change it now, yes, they just say you have to take two breaths in the morning, and they do it regularly if I have felt worse for a little while. I’ll just get more, double up.

M2: Yes, I feel very safe.

When it comes to medication, the informants trust the guidance of the digital nurses because they feel it is their responsibility to adjust their medication. They feel secure knowing that others are assisting them and providing direction with their medication management.

When the RNs oversee the health condition of the informants, their independence in managing their chronic disease and their understanding of their medication do not seem to improve. It appears that the RNs are still somewhat paternalistic. However, the informants do experience a sense of safety, particularly when it comes to their health and medication.

### Value Creation

#### Behavior

This category identifies the former daily routines that had to change or be excluded from the informants’ lives because of COPD. Furthermore, it highlights the new routines that should be adapted because of the weakened ability caused by their condition.

The informants must adapt their daily routines to accommodate their diminished capacity because of COPD, necessitating the establishment of new habits. Consequently, they may take fewer walks, experience reduced appetite, or sleep longer than usual. This limitation often confines the informants to their residences. When queried about their daily lives, the informants provided a range of responses. Some spoke very briefly and exhibited a negative attitude, while others believed that obtaining a comprehensive understanding of how COPD impacts daily activities was crucial.

Interviewer: ...oh if you should tell me how a typical everyday looks for you K1, what do you do on a general day?

W1: Sitting here

Interview: You sit there

W1: Yes (cough), but sometimes when I’m well, I go out in the garden.

In this case, the informant primarily spends time sitting on the same couch and does not elaborate much on her everyday activities. She finds joy in moving outside and into the garden whenever possible. Previously, she engaged in various artistic activities and housework as part of her daily routine, but these tasks are no longer feasible due to her health.

Some informants expressed that it was still important to maintain cleanliness in their own homes. While the municipality provides cleaning assistance to the majority of the informants, some individuals still prefer to handle specific tasks on their own.

W2: ...I said to her, now don’t think I’m crazy but I’ve been standing and ironed my bedsheets for several days, then she was about to faint (...). Only the elderly irons their bedsheets. I have always done it, and I will not stop doing it as long as I can stand on my feet.

She continues to prioritize tasks such as making her bed and changing the sheets, as she has always done. Despite the challenges posed by her health, she decides to persist because these tasks hold significant importance for her. However, she acknowledges that it may take several days to complete them. By contrast, most of the informants expressed overall dissatisfaction with the cleaning assistance provided by the municipality, stating that they often had to make numerous corrections.

Another aspect they felt had changed because of their condition was the rhythm of the day. They found that getting out of bed in the morning was becoming more challenging, or they noticed that they were waking up earlier. This change could be attributed to their increased frequency of sleeping and reduced engagement in everyday activities.

M1: Yes. Well, but I wake up before Satan gets his shoes on, because I am used to doing something, and I cannot really more, so I never get really really tired (coughing), so I do not get so terrible many hours of sleep (coughing).

Here it is highlighted that the informant’s daily rhythm has shifted from its previous pattern, and the indication is that their lack of sleep stems from both reduced activity and diminished tiredness. None of the informants mentioned experiencing anxiety or shortness of breath during the night, which could also contribute to a different daytime rhythm. However, the increased need for sleep during the day was frustrating, as it could result in missing out on certain activities.

#### Hobbies

The informants engaged in different activities in their lives that held personal value for them, and some had to alter their activities because of COPD. This category focuses on the activities that the informants currently undertake and have previously engaged in.

The activities and interests of the informants varied depending on their weekly or daily routines. However, their condition often took precedence over their interests, and the activities that were feasible differed among the informants. Additionally, there was a gender disparity, with men favoring fishing and other outdoor activities, while women tended to engage in activities such as handicrafts.

M2: So now that I have been sick, yes, I go out and fish a little, then I go and help a little with some horses.

W3: ...and then I’m knitting or doing the crosswords or trying to sew on the sewing machine (laughing).

M1: yeah, I’m trying to do the things I care about and like, unfortunately I can’t paint anymore, as I cannot stand the smell of turpentine anymore, it’s sad because I’ve spent a lot of time painting, I don’t have the energy to start writing more books. So I, I read a little, it’s a bit difficult now with these glasses, but I’ve read a lot, and I get a lot of pleasure from it.

The different accounts provided by the informants offer insights into how interests are possible for individuals with chronic illness as well as how the condition can prevent them from pursuing activities they like/enjoy. Despite certain interests being curtailed by the condition, informants still strive to engage in activities that bring them joy and hold value for them. However, in some cases, informants found it challenging to pursue their interests because of the awareness of potential shortness of breath.

The informants expressed similar daily desires and willingness to go outside, but their walks had become shorter over time. Occasionally, they cited the weather as an excuse to stay indoors.

W2: Then the little dog and I go in and rest for an hour or half an hour, and lull a little and sleep a little. Perhaps it is something completely different. Then I get up and get ready, and then I go with my little dog and pick up the newspaper in the mailbox. We used to go for longer walks, but I don’t unfortunately, I can’t do it anymore.

W3: For just such a trip, so there is not much nature to go and look at from here and down to the municipal office, but just to get out and get some fresh air

The informant acknowledges here that the challenge of going on longer walks is something that annoys her, but she simply cannot manage it anymore due to shortness of breath. Despite this limitation, going for a walk can bring relief to the informants. Even though nature may not always be visually stunning, the informant finds solace in being able to get outside, especially on slightly gloomier days.

#### Motivation

This category underscores how the informants experienced a lack of motivation to engage in daily activities. Throughout the conversations, there was a tendency to discuss things they would love to do but lacked the competence or strength to accomplish, or they invented excuses because the tasks seemed overwhelming. These could range from simple tasks such as planting a rose to more complex endeavors such as writing a book, attending gym classes, or cooking.

W1: Not at all, and then you lose the motivation.

Interviewer: Yes, I can understand, if you have been somewhere where you think it was good, and then you come to something else that you don’t think is at the same standard...

W1: but then, okay, I am not...there, but oh its hunting me, when I have to go. I will probably just get it over with, right?

The informant’s desire and motivation to participate in a COPD exercise team depend on how the teacher conducts the sessions. A negative experience with teaching methods in the past has diminished the informant’s motivation, making it difficult for them to participate. It has transformed from an activity that brings joy to feeling like an obligation, something that the informant feels they must do rather than something they want to do.

### Resources

#### Social Networks

This category explores the social networks that the informants are a part of and how they use them in their day-to-day activities.

The size of the social network varied among the informants, but the significance of social interactions was equally important to all of them. Family relationships showed considerable diversity among the informants, with some maintaining close contact with their family through daily conversations, weekly scheduled visits, or having their spouse present. By contrast, there were some informants who had limited contact with their families and spent much of their daily lives alone.

W2: I miss him very much. We lived in Fyn, and we talked over the phone several times a week. I miss my family very much, and I also miss my friends. And they all passed away...

W2: ...I talk to my daughter. So so I don’t talk about..., she can say to me, I think you sound a little stalled mother, because then I’m just for the moment and then, and then we’re not talking about it anymore. She knows what it is, but we don’t need to.

The above description indicates that the informant is alone due to deaths in the family and social circles, and there are a significant number of people missing from her family. Interestingly, the informant does not mention at the present time that she still has 2 daughters, which she only brings up later in the interview. The informant’s description of the varying family relations indicates that she is left more isolated and alone, which telephone conversations with her daughters cannot fully mitigate. The conversations surrounding the family and friends of the “lonely” informants were marked by a sense of sadness and depression over the lack of contact.

Informants with close family relations expressed how their family and close relationships maintain continuous contact with them. The conversations were even interrupted by phone calls from their families, highlighting the frequent and ongoing nature of their communication.

M2: Yeah, she is calling, or she has stopped a little, but otherwise she calls every morning, around 9 o’clock or something like, “How do we breathe today?” She says then, (laughing)

W1: Then I also have my granddaughter, I talk a lot with her, but I also take care of what I said to her, because she is a little unstable.

There are 2 different scenarios for contact described here, both indicating that the informants have contact with their families, signifying close relationships. In one instance, it is the informant’s mother showing interest in their self-monitoring and health status. In the second scenario, the informant not only maintains close contact, but also plays a protective role for her grandchild, who also suffers from a diagnosis. Despite varying family dynamics, it is evident that the informants can be divided into 2 groups: those with close family relations and those lacking such connections.

Their interest in engaging in social activities was also significant, but the informants often found themselves coming up with excuses for not participating or found it challenging to leave their homes.

W3: Yes, I haven’t reached it yet, but I’m probably getting enough. There has been something on Friday, because otherwise I had set myself up for, I have otherwise gone to gym down in Vig, but.. that, which is quite far from the station off and down to Balsagård (...), of course I can go down there from time to time, but as I have it, oh for the last season there, I was not there quite many times, but it costs no matter what, they do not pull anything from because I have not been there, and that.. it annoys me a little. Then there is Red Cross that has something like this in high town.. exercise, sport is known enough, and it is every Friday morning, but there has been something here the last couple of Fridays, and I also have to just get into the rhythm that I have to go there until half 10 p.m.

As emphasized, the informant highlights that traveling a long distance to attend gymnastics is a major obstacle. Despite continuing to pay for it, this does not motivate her to attend regularly. She also mentions the challenge of incorporating it into her Friday routine and making it a regular part of her schedule.

They did not envision themselves participating in social events related to their COPD. Some of them were members of Lungeforeningen, the Danish Association for Lung Diseases. Although the Lung Association organizes various gatherings for those with COPD, none of them appeared interested in attending.

#### Health Professionals

The category focuses on the informants’ interaction with various health professionals and how those relationships hold significance for them.

The relationship with health professionals was highly significant, as it was essential for the informants to feel secure while also being with mutual respect and seriousness. The informants interacted with various health professionals in their daily lives, and this analysis distinguished the difference between “ordinary” health professionals and PreCare nurses.

The informant’s relationship with the assistance offered is crucial.

W2: After a hospitalization for yeah I don't know, let's just say a year ago. There seems to be, I can’t remember who thinks that there should be a home care and dosing the medicine. And now you must not misunderstand me because I am not a racist, but there comes a little colored girl who could not really speak Danish and she was not very sweet if she had been sweet and smiling, pleasant, then it would have been something else, but it was she not, and then she made mistakes twice after the other, and I discovered it quickly, and it is not, it isn’t the tablets we sit and play with.

According to the informant, the connection with home care has been challenging because of mistakes and uncertainty. As a result of this, the informant has lost faith in the home care, which should be there to lend a helping hand and not cause her problems on a regular basis.

Unlike other health care professionals, the nurses in PreCare have succeeded in establishing a sense of security and mutual respect with the informants. The collaboration with those involved in their COPD care instills confidence in the PreCare project and the nurses among the informants.

M1: There is most of the contact through the nurses, just to start, just when I started up there was a doctor who was here, and so I have nothing bad to say about him, oh and it is also those who prescribe some medicine if I lack it, and such some things not too (slang). I think I have a good relationship with them, and are really pleased to have them, oh...and feel there is a great confidence to be with them. So, as I said earlier, I was sure I would have become a burden for the hospital if I had not known them. The society saves money, and that’s not bad.

W2: But Nærklinikken has helped me, exceptionally. I’m glad I got in touch with you, you can believe. I don’t know what I would have done without medical care. They do nothing.

The informant expresses happiness for the nurses and thinks that their connection is good. The following description includes several elements though. The informant mentions that their participation in PreCare makes them feel like less of a burden for the hospital and the municipality, which holds significant meaning for them. He also expresses faith in the nurses, which he had expressed several times in the conversation. Being a part of a project that highlights the superior care provided by nurses compared with general medical care has been particularly significant for the female informant.

Nurses are not only available to informants, but also offer support if an informant’s condition deteriorates. When an exacerbation happens, trust means the informant has no reason to question the nurses and takes the prescribed prescription without a second thought.

M2: Yes, Nærklinikken says it, it is the ones who change it now, yes, they say you just have to have two breaths in the morning, and they do it regularly if I have had it a little bad for a while. No, then I’ll just be put up, double up.

Without hesitation, the informant chooses to follow the nurses’ recommendations. He has completely surrendered to the project, giving them full responsibility for his condition.

#### Technology

This category covers the informants’ daily technology and their search for health-related information.

All informants admitted to having technology at home, although the way they used it and its purpose varied. For some, technology provides entertainment during moments of boredom in their daily lives.

W1: Yes, I’m mostly on the computer when I get bored.

Interviewer: What are you doing on the computer?

W1: I am playing games

W1: I have two different games I have discovered.

Technology was not utilized by the informants as a means to gather information about COPD. The informants felt they already possessed all the information they needed about COPD, and they were concerned that obtaining more information might increase their anxiety.

M1: It is very very rare; it is very rare. If I happen to hear that there is something new about it, then I can well find out to look it up, that it is not so exciting to read about, so

W1: I think the more you read, the more nervous you become.

The informants do not use technology to seek information about their condition. The informants did not mention being part of online groups where information could be shared during the interviews. None of the informants mentioned using social media platforms such as Facebook as a community for sharing information about their condition. They also emphasized that they generally did not share information about their condition through digital solutions.

M3: It irritates me sometimes when we sit, sometimes I cut through and say, now we don’t want to talk about illness, because, oh, then such a short evening can go

The medical equipment provided by PreCare did not pose any problem for the informants to use on a daily basis. They all expressed how easy it is to use it and how it takes only a few seconds to use the technology. They appeared confident and stated that they performed the measurements every day.

M4: it’s so easy, that’s in order...the only thing is now just, the crazy computer goes out, or (...), I can’t restart, even though I have PIN code...no matter what I do, it won’t, so I wait when it comes a past...so it can restart again, the only problem...

Despite the ease, they experienced some issues with the devices. The informants encountered issues with logging in, forgot to charge the tablet, or even misused the thermometer.

## Discussion

### Principal Findings

This study offers valuable insights into understanding the needs, values, and preferences of individuals living with COPD as well as which communities they identify with in a digital context. Indeed, the findings highlight that while fluctuations in their health condition significantly affect the daily lives of the informants, factors such as having hobbies, old habits, and social connections play a crucial role in their overall well-being. This underscores the importance of recognizing individuals with COPD as complete human beings beyond their medical condition.

It is interesting to note the distinction between the 2 communities the informants belong to. The community centered around the RCC represents a vital support network for them, where they feel included and have developed trust with the staff. This highlights the importance of such digital health services in providing continuous support and guidance for individuals managing chronic conditions such as COPD. Involving close relatives in the community centered around the RCC can further enhance the support system for individuals with COPD. The other community involves participation in social activities outside the context of their condition which provides informants with opportunities for social interaction, enjoyment, and connection with others beyond their health concerns. These activities offer a sense of normalcy and contribute to their overall quality of life, allowing them to engage in meaningful relationships and experiences beyond the realm of COPD management. Even though the informants may face constraints due to their condition, they still find value in participating in social activities, even if their involvement is limited.

### The Needs, Values, and Preferences in the Digital Context

The informant’s emphasis on maintaining their daily activities underscores the significance of preserving their sense of normalcy and independence despite their COPD symptoms. It reflects their desire to continue living fulfilling lives and not be defined solely by their health condition. Symptoms often contribute to a lower quality of life and well-being. The findings from an earlier study [32] resonate with the experiences reported by individuals with COPD in this study. Breathlessness, a common symptom of COPD, can significantly impact an individual’s quality of life by limiting their ability to engage in daily activities and causing distress. This aligns with the participants’ reports of reduced quality of life related to breathlessness, highlighting the importance of addressing this symptom to improve overall well-being for individuals living with COPD. While outdoor activities may be affected by COPD, the focus of the informants seems to be more on how the disease impacts their ability to engage in everyday tasks and maintain their hobbies or household chores. This also resonates well with another study including interviews of patients with COPD [7]. The study revealed that for women, being active in housekeeping was important and valued, while for men, maintaining the garden held similar significance. They also reported that people with COPD prefer activities within their home or immediate vicinity, showing little interest in engaging in social activities located far away due to their reduced physical abilities. The need to be close to a safe environment was similar for some of the informants in this study. However, some reported that they did leave their house. For example, one informant was helping with some horses, while others needed to take their dog to the dog groomer. Access to virtual support may play an important role here, as the informant could call the RN in the RCC at any time if anything were to happen.

### The COPD-Related Conditions’ Impact on the Identity

For the informants, it was important to avoid discussing or being associated with their COPD condition by shifting the topic during the interviews. This aligns with a previous study where people with COPD were not interested in being identified solely by their illness [33]. They found that their identity related to their condition, termed “illness identity,” was affecting their roles and could potentially separate them from the social network or community. An additional contributing factor to social segregation was a sense of having a self-inflicted disease, leading to feelings of shame and guilt [33]. However, this was not evident in our data.

Most, but not all, of the informants appear to be able to cope with their diagnosis and condition. They try to continue the same kind of interactions and activities while considering their condition’s restrictions. As a result, individuals experience a sense of maintaining their own identity within their communities, yet occasionally feel the sense of lacking something. This can involve engaging in distant activities, such as joining a choir.

### The Two Communities of Practice

#### The Community of COPD Practice

In relation to the community with its formal caregivers, the informants experienced a genuine interest from the RNs in their well-being and they provided them with support in an empathic way. This experience may be attributed to the PreCare environment, with free access to RNs 24/7, where they always kept an eye on the informants. This may explain the absence of “anxiety” in the interviews with the informants. These could be attributed to the RN’s *ability* to provide immediate support in response to changes in their health condition, with medication for deterioration accessible at home [32]. Anxiety, which often dominates the daily lives of individuals with COPD, is thus better managed [32]. Therefore, it is necessary for the RNs to be trained to instill confidence in individuals with COPD or other LTHCs, enabling them to feel more independent and socially active with the RCC, their equipment, and a medicine box readily available, thereby reducing anxiety and maximizing the benefits of their resources.

The informants’ immediate access to the RNs in the RCC appears crucial, as it enhances their self-efficacy and confidence in how their equipment aids them in managing deterioration.

However, despite feeling secure in their use of the equipment, the informants did not appear to be influenced by their ability for self-management and did not feel more empowered, likely due to experiencing the RNs as being paternalistic. The informants were unable to fully benefit from the virtual support environment due to the influence of the RNs and instead remained in a passive role.

RNs and other clinicians will need to be aware of how they communicate to facilitate a dialog that is not experienced as paternalistic, but rather as a coaching conversation.

#### The Community of Social Practice

In relation to their social communities, the extent of social relationships varied among the informants. The importance of a family community aligns with the findings of Nicolson and Anderson [32], who showed that family and relatives significantly influence the quality of life for people with COPD. Nicolson and Anderson [32] identified that COPD impacted how individuals connected with relatives and perceived their ability to fulfill their roles within the family. In contrast to the study by Nicolson and Anderson [32], our findings indicate that the informants’ family roles were not influenced by their COPD. They maintained their roles and continued normal interaction with their relatives and families.

Not all informants participated in social activities or were part of a local community. Those without support from friends and families experienced difficulties engaging in and finding motivation for social activities. They felt lonely when left alone in their homes, whereas those with a social network experienced loneliness to a lesser degree. This contrasts with another study [7], which found that loneliness was also a major issue for those with family support, such as spouses and friends [7]. Those who felt lonely because of their lack of participation in social activities found some comfort in the availability and contact with the RN, which to a certain extent reduced their experience of loneliness.

This underscores the necessity for RNs to possess skills in mental and social support, which should be included in the education programs for nurses.

Online resources such as patient portals and social media (eg, Facebook) can constitute a community for people with COPD. However, despite the availability of these platforms to our informants and their daily use of tablets, none of them considered these online opportunities in relation to their COPD condition. This may be due to various reasons. Some informants felt they had sufficient knowledge or were unsure how to interpret the overwhelming information on the internet. Additionally, they may have wanted to avoid exposing their diagnosis or involving others outside their close network [34]. The role of the PreCare environment and the RNs may substitute the need for a social media platform or COPD-related conversations on platforms such as Facebook.

When the informants do not participate in online communities, they may miss the opportunity to access new knowledge or learn from others with similar conditions. This lack of engagement can reduce their ability to manage their condition effectively and hinder their empowerment. Joining an online community and being actively involved can help transform newcomers into “super users” and “experts” [35]. These “online experts” can then help other members of the community, forming a virtual community of practice [25]. Thus, participants in the PreCare project may miss the opportunity to develop into experts through online activities but may instead develop this competence through participation in other communities or collaboration with the RNs.

### Limitations

The study is based on 7 informants. This may be considered a limitation, as the relatively small number may result in some perspectives of people living with COPD not being expressed in the data. However, as all informants are exposed to the same PreCare environment, have the same diagnosis of COPD, and live within the same area, we find that the necessary number of participants to have enough power of information is met [36]. This is supported by the presence of common patterns among the informants and the alignment of the overall findings with the data obtained in the PreCare project. Further studies are needed to confirm our findings before they can be considered valid for scaling up and evaluating the impact of working with landscapes or communities of practice. This support aims to foster a sense of more active and independent living based on existing values.

### Perspective

The findings suggest that education for RNs and other health professionals should focus on their roles as professionals while also acting as facilitators. They should avoid being paternalistic to create a space for the development of self-efficacy and self-management. A motivating factor will help develop self-efficacy and confidence, enabling people with COPD to be more socially active and encouraging them to pursue their desires. Health professionals play a key role here, as they can provide the means to help individuals become more active, thereby increasing their well-being.

### Conclusions

When using digital health solutions, people’s needs, values, and preferences should be considered, focusing primarily on addressing the whole person rather than just the “illness.” This approach creates the best opportunity for individuals to maintain their daily activities and feel empowered. The 2 communities the informants take part must work together and will intersect in their daily lives. They should support each other, involving the needs, values, and preferences of the individuals, and ensuring that upcoming digital health services include and embrace situated learning to enhance people’s empowerment and self-management. Furthermore, new educational programs should be developed or considered to enhance the competencies of RNs who are involved in digital health services. This will provide the best opportunity for the 2 communities to collaborate and support the daily activities of people with chronic conditions.

## Appendix

Multimedia Appendix 1 Interview guide.

Multimedia Appendix 2 Categorization of the data.

## Abbreviations

COPD: chronic obstructive pulmonary disease
ECM: Epital Care Model
GOLD: The Global Initiative for Chronic Obstructive Lung Disease
LTHC: long-term health condition
RCC: response and coordination center
RN: registered nurse
