# Supplementary material for: Preferences, Needs, and Values of Patients With Chronic Obstructive Pulmonary Disease Attending a Telehealth Service: Qualitative Interview Study
Source: JMIR Hum Factors. 2024 Jun 21;11:e53131. doi: 10.2196/53131 (PMC11226923; doi:10.2196/53131)
Supplement: Multimedia Appendix 1 [file humanfactors_v11i1e53131_app1.docx]

| Themes | Help Questions |
| --- | --- |
| Daily | Can you tell me what a typical day-to-day looks like for you?   - What have you done in the last week? - What activities do you undertake daily? Week? - How do you handle the general household? –cleaning, laundry, shopping and cooking? - Are there any things you do less or not do because of your COPD? - What does an atypical day look like for you? |
| Diagnoses | Can you tell me how COPD has affected your life?   - How has COPD affected your life? - What changes has COPD made to your activities? - How do you manage your medication?   - New medication   - Side effects   - Amounts   - Exacerbations - How do you know how much of the prescribed medication you should take? |
| Measurements | Can you tell me when and how you are doing your measurements?   - Where and when do you take the equipment from PreCare with you? - How is it to use the equipment from PreCare? |
| Communities | Are you involved in any kind of social activities?   - Whom in your everyday life do you talk to about your COPD? - Where and how do you share your experiences with COPD? - Whom do you trust to share your information with? |
| Nærklinikken/PreCare | Can you tell me about your participation in the Nærklinikken/PreCare?   - How is your contact with Nærklinikken/PreCare?   - In relation to medication?   - Advice about other factors   - Degrees of confidence in the Nærklinikken/PreCare |
| Competences and digital literacy | How do you use technology in your everyday life?   - How do you use it in relation to your health? - Do you use any other technology yourself to monitor your COPD than the one you have received from Nærklinikken/PreCare? - How does it help you? - How do you get knowledge about your COPD? |

**Interview guide for the ethnographic interview**
This interview guide is based on James P. Spradley and the questions below are based on his methods. The questions are defined to fit the theory. The questions is focusing on the informants managing and handling their COPD, including communities and technology. The goal of this interview guide is that it should help to keep a friendly and informal conversation between the participant and the Ph.D. student.
